# The genetic correlation and causal association between key factors that influence vascular calcification and cardiovascular disease incidence

**DOI:** 10.3389/fcvm.2023.1096662

**Published:** 2023-01-26

**Authors:** Xiaolin Ni, Lei Liu, Yao Yao, Chi Zhang, Huabin Su, Yuan Lv, Rongqiao Li, Liang Sun, Qi Zhou, Xiaoquan Zhu, Ze Yang, Zuoguan Chen, Wei He, Huolan Zhu, Shenqi Zhang, Caiyou Hu, Huiping Yuan

**Affiliations:** ^1^The Key Laboratory of Geriatrics, Beijing Institute of Geriatrics, Institute of Geriatric Medicine, Chinese Academy of Medical Sciences, Beijing Hospital, National Center of Gerontology of National Health Commission, Beijing, China; ^2^State Key Laboratory of Stem Cell and Reproductive Biology, Institute of Zoology, Chinese Academy of Sciences, Beijing, China; ^3^Institute for Stem Cell and Regeneration, Chinese Academy of Sciences, Beijing, China; ^4^Department of Medical Microbiology and Infection Prevention, University Medical Center Groningen, University of Groningen, Groningen, Netherlands; ^5^China Center for Health Development Studies, Peking University, Beijing, China; ^6^Jiangbin Hospital, Zhenjiang, China; ^7^Department of Vascular Surgery, Beijing Hospital, National Center of Gerontology, Institute of Geriatric Medicine, Chinese Academy of Medical Sciences Peking Union Medical College, Beijing, China; ^8^Department of Cardiology, Beijing Hospital, National Center of Gerontology, Beijing, China; ^9^Department of Geriatrics, Shaanxi Provincial Clinical Research Center for Geriatric Medicine, Shaanxi Provincial People’s Hospital, Xi’an, China; ^10^Department of Joint and Sports Medicine, Zaozhuang Municipal Hospital Affiliated to Jining Medical University, Zaozhuang, Shandong, China

**Keywords:** serum calcium, vitamin D, vitamin K, cardiovascular disease, risk factor

## Abstract

**Background:**

Serum calcium (Ca), vitamin D (VD), and vitamin K (VK) levels are key determinants of vascular calcification, which itself impacts cardiovascular disease (CVD) risk. The specific relationships between the levels of these different compounds and particular forms of CVD, however, remain to be fully defined.

**Objective:**

This study was designed to explore the associations between these serum levels and CVDs with the goal of identifying natural interventions capable of controlling vascular calcification and thereby protecting against CVD pathogenesis, extending the healthy lifespan of at-risk individuals.

**Methods:**

Linkage disequilibrium score (LDSC) regression and a two-sample Mendelian randomization (MR) framework were leveraged to systematically examine the causal interplay between these serum levels and nine forms of CVD, as well as longevity through the use of large publically accessible Genome-Wide Association Studies (GWAS) datasets. The optimal concentrations of serum Ca and VD to lower CVD risk were examined through a restrictive cubic spline (RCS) approach.

**Results:**

After Bonferroni correction, the positive genetic correlations were observed between serum Ca levels and myocardial infarction (MI) (*p* = 1.356E–04), as well as coronary artery disease (CAD) (*p* = 3.601E–04). Negative genetic correlations were detected between levels of VD and CAD (*p* = 0.035), while elevated VK1 concentrations were causally associated with heart failure (HF) [odds ratios (OR) per 1-standard deviation (SD) increase: 1.044], large artery stroke (LAS) (OR per 1-SD increase: 1.172), and all stroke (AS) (OR per 1-SD increase: 1.041). Higher serum Ca concentrations (OR per 1-SD increase: 0.865) and VD levels (OR per 1-SD increase: 0.777) were causally associated with reduced odds of longevity. These findings remained consistent in sensitivity analyses, and serum Ca and VD concentrations of 2.376 mmol/L and 46.8 nmol/L, respectively, were associated with a lower CVD risk (*p* < 0.001).

**Conclusion:**

Our findings support a genetic correlation between serum Ca and VD and CVD risk, and a causal relationship between VK1 levels and CVD risk. The optimal serum Ca (2.376 mmol/L) and VD levels (46.8 nmol/L) can reduce cardiovascular risk.

## Introduction

The rate of global population aging continues to accelerate (1), contributing to elevated risks of a range of age-related disorders and diseases that ultimately impair function and increase the risk of mortality (2). The World Health Organization (WHO) has established cardiovascular disease (CVD) as the most prominent global cause of death, contributing to 17.9 million deaths per year on average (3). Epidemiological research has revealed a range of factors that are related to CVD risk, including nutrient intake, alcohol consumption, exercise, and smoking (4–8). The ability to prevent CVD and to facilitate a healthier aging process is thus strongly dependent on the identification and mitigation of early CVD-related risk factors.

In recent work, vascular calcification has been identified as a common finding in patients with various forms of CVD including atherosclerosis, coronary artery disease (CAD), myocardial infarction (MI), heart failure (HF), and ischemic stroke (IS), suggesting a possible relationship between calcium (Ca) deposition and these conditions (9–12). In addition, serum concentrations of Ca, vitamin D (VD), and vitamin K (VK) are closely related to vascular calcification incidence, ultimately impacting CVD development. In particular, elevated serum Ca has been shown to contribute to direct increases in vascular calcification and CVD risk. However, many prior studies assessing the relationship between CVD and serum Ca levels have yielded inconsistent findings (13).

Vitamin D plays an important role in regulating the endocrine system and whole-body Ca homeostasis (14), with VD deficiencies contributing to a range of CVD risk factors and higher mortality rates among CVD patients (15). Even so, recent randomized controlled trial data suggests that VD supplementation does not offer any benefit with respect to CVD (16). There is thus a clear need for further research aimed at clarifying the nature of any protective benefits provided by VD in CVD. VK similarly functions as a key regulator of Ca homeostasis, impacting the cardiovascular system *via* activating matrix Gla protein, which can prevent calcification. When inactive, this protein is associated with a range of CVD-related risk factors including increases in insulin resistance, vascular calcification, valvular calcification, arterial stiffness, and HF indices that all contribute to higher rates of CVD-related death (17). However, definitive population-level causal evidence regarding the relationship between VK and CVD is currently lacking.

Therefore, based on the direct and indirect effects of serum Ca, VD, and VK concentrations on vascular calcification, and the fact that vascular calcification has become a common cause of various types of CVD, in this study, single nucleotide variant (SNV)-based genetic correlation analyses and a two-sample Mendelian randomization (MR) framework were leveraged to conduct a comprehensive analysis of the causal relationships among serum Ca, VD, VK, and a range of CVD outcomes [including CAD, MI, HF, atrial fibrillation (AF), all stroke (AS), all IS (AIS), small vessel stroke (SVS), large artery stroke (LAS), and cardioembolic stroke (CES)]. In addition, CVDs and longevity are in essence the result of interaction between genetics and environment. Studies have shown that different alleles of the same gene locus affect homeostasis of vascular microenvironment through regulation of expression, which may lead to two opposite outcomes: CVDs and longevity (18). Longevity and CVD are both interconnected and opposites (19). Therefore, in our study, besides the normal control, longevity was also used as a negative control to compare with CVD. The goal of these analyses was to identify interventions with the potential to reduce the morbidity or mortality associated with CVD, contributing to healthier aging and a longer life (2).

## Materials and methods

### Study design

Linkage disequilibrium score (LDSC) regression analyses enable the examination of SNV-associated heritability and coheritability between traits. MR analyses permit the evaluation of possible causal relationships between two traits based upon Mendel’s law of independent inheritance, offering an opportunity for a natural randomized control trial (RCT) (20). LDSC and MR approach complement one another as strategies for exploring how to traits are related to one another. A restrictive cubic spline (RCS) strategy was also used with appropriate multivariate regression analyses as a means of examining relationships between exposures and outcomes to define optimal threshold values for exposures of interest (21) (Supplementary Figure 1). As the analyses performed herein were based upon publically available datasets, no further ethical oversight or informed consent were necessary.

### Outcome data source

The primary outcomes for this analysis were CVDs and longevity, with outcome data sources being provided in detail in Table 1. AF-related data were derived from a study performed by Nielsen et al. (22), with paroxysmal or permanent AF and atrial flutter included in the definition of AF (60,620 cases, 970,216 controls), while summary statistics for HF were derived from the largest published Genome-Wide Association Studies (GWAS) meta-analysis performed by the HF Molecular Epidemiology for Therapeutic Targets (HERMES) Consortium analyzing individuals of European ancestry (47,309 cases, 930,014 controls) (23). Participants in this study were individuals diagnosed with HF of any etiological basis determined based upon left ventricular ejection fraction (LVEF) (24). Summary-level CAD data were derived from the CAS Genetics (CARDIoGRAMplusC4D) Consortium (122,733 cases, 424,528 controls) (24). Stroke summary statistics for individuals of European ancestry (67,162 cases, 454,450 controls) including 67,162 AS, 60,341 AIS, 6,688 LAS, 9,006 CES, and 11,710 SVS cases, were derived from the MEGASTROKE consortium aimed at reducing bias resulting from population stratification (25). Summary-level MI data were derived from the CARDIoGRAMplusC4D (60,801 cases, 123,504 controls), MIGen, and CARDIoGRAM Exome consortia (42,335 cases, 78,240 controls), and ESP EOMI (4,703 cases, 5,090 controls) datasets (26).

**TABLE 1:** Genome-Wide Association Studies (GWAS) data sources.

Phenotypes	GWAS data source	Sample size	Ancestry	Covariates	Objective
Calcium	UK Biobank (UKB) (26)	313,387 Individuals	European	Genotype principal components (PCs) (the top 40 PCs of the UK Biobank-provided genotype-based global PCs), age indicator variables (one for each integer age), sex, 5-year age indicators by sex interactions, self-identified ethnicity, self-identified ethnicity by sex interactions, fasting time (one indicator per fasting time, except a single indicator for >18 h and for 0 or 1 h), estimated sample dilution factor (icosatiles), assessment center indicators, genotyping batch indicators, icosatiles of time of sampling during the day, month of assessment (indicators for each month of participation, with the exception that all of 2006 and August through October of 2010 were assigned a single indicator), and day of assay (one indicator per day assay was performed).	Exposure (LDSC regression, MR, and RCS)
Vitamin D	UK Biobank (UKB) (27)	417,580 Individuals	European	Age at time of assessment, sex, assessment month, assessment center, supplement-intake information, genotyping batch and the first 40 ancestry PCs as covariates.	Exposure (LDSC regression, MR, and RCS)
Vitamin K1	CHARGE Consortium Nutrition Working Group cohorts (28)	2,138 Individuals	European	Age, sex, and study-specific covariates, including population stratification by PC analysis (PCA) and clinical site.	Exposure (LDSC regression and MR)
AF	The Nord-Trøndelag Health Study (HUNT), deCODE, the Michigan Genomics Initiative (MGI), DiscovEHR, UK Biobank, and the AFGen Consortium (20)	60,620 Cases and 970,216 controls	European	Including covariates birth year, sex, genotype batch, and PCs 1–4.	Outcome (LDSC regression and MR)
HF	Heart Failure Molecular Epidemiology for Therapeutic Targets (HERMES) Consortium (21)	47,309 Cases and 930,014 controls	European	All studies included age and sex (except for single-sex studies) as covariates in the regression models. PCs were included as covariates for individual studies as appropriate.	Outcome (LDSC regression and MR)
CAD	CARDIoGRAMplusC4D and UK Biobank (UKB) (22)	122,733 Cases and 424,528 controls	European	age, gender, the first 30 PCAs	Outcome (LDSC regression and MR)
Stroke	MEGASTROKE Consortium (23)	67,162 Cases and 454,450 controls, cases including 67,162 AS, 60,341 AIS, 6,688 LAS, 9,006 CES, 11,710 SVS.	European	NA	Outcome (LDSC regression and MR)
MI	CARDIoGRAMplusC4D, MIGen and CARDIoGRAM Exome consortia, and ESP EOMI datasets (24)	Interrogated the CARDIoGRAMplusC4D (60,801 cases, 123,504 controls), the MIGen and CARDIoGRAM Exome consortia (42,335 cases, 78,240 controls), and ESP EOMI (4,703 cases, 5,090 controls) datasets	European	NA	Outcome (LDSC regression and MR)
Longevity	The previously published GWA studies on longevity (25)	11,262 cases and 25,483 controls	European	NA	Outcome (LDSC regression and MR)

AF, atrial fibrillation; HF, heart failure; CAD, coronary artery disease; MI, myocardial infarction.

Longevity analyses were performed with summary statistics derived from a recent GWAS meta-analysis of individuals of European ancestry in ∼20 population- or family based cohorts in Europe and the USA (27). Cases (*n* = 11,262) were individuals who lived to an age above the 90 or 99th percentile age based on cohort life tables from census data for the appropriate country, sex, and birth cohort. Controls (*n* = 25,483) were individuals who died at or before the 60th percentile age or whose age at the last follow-up visit was at or before the 60th percentile age.

### Data sources and variant selection

Data pertaining to serum Ca (28), VD (29), and VK (30) concentrations for the included sources of exposure data are summarized in Table 1. Serum Ca and VD levels were derived from the UK Biobank Resource (Project #73697). All participants in the UK Biobank had provided informed consent, with oversight from the North West Multi-Centre Research Ethics Committee (11/NW/0382). VK1-related data were derived from the Cohorts for Heart and Aging Research in Genomic Epidemiology (CHARGE) Consortium Nutrition Working Group (30). The largest GWAS study focused mainly on individuals of European ancestry was used to select genetic variants related to modifiable risk factors.

Instrumental variables for modifiable risk factors were determined with the Plink software through the use of the clump procedure. Considering that VK did not screen out independent sites at the threshold *p* < 5E–08, single nucleotide polymorphisms (SNPs) linked to risk factors were selected at the selected threshold for possible genome-wide significance (*p* < 1E–05). While for serum Ca and VD, the causal correlation didn’t change much when we used more stringent threshold (Supplementary Table 2). When linkage disequilibrium (*r*^2^ > 0.1) for SNPs was evident for a given trait, SNPs that were most strongly associated with the exposure of interest based on the smallest measured *p*-value were selected. SNPs not included in CVD- or longevity-focused GWAS datasets were not included in this study. For the selected outcomes, the number of SNPs chosen as instrumental variables ranged from 8 to 448. These variants explained from 0.021 to 0.529% of phenotypic variation (Supplementary Table 3). To synchronize data between exposure and outcome GWAS, estimates of SNP effects were flipped with unrelated alleles and effects.

### LDSC regression analyses

Cross-trait LDSC analyses were used to evaluate genetic correlations between pairs of phenotypes and genome-wide SNPs (31). LD scores for individual SNPs were determined in accordance with genotypes for common SNPs [minor allele frequency (MAF) > 0.01, Hardy–Weinberg equilibrium *p* > 1 × 10^–5^] over a 10 Mb window when evaluating data derived from 503 European individuals included in the 1000 Genomes Project. The exact number of SNPs used in the genetic correlation analyses in each pair were shown in the Supplementary Table 1. LDSC analyses were then performed through the use of a weighted linear model *via* regressing *Z*-statistic products for two traits on LD scores across all variants throughout the genome. The resultant regression slow should provide an unbiased tool for estimating genetic correlations even if some individuals overlap between two GWASs. Bonferroni correction was used to correct for multiple testing, with a two-sided significance level of 0.0056 being established (0.05 divided by the nine included outcomes). Those associations exhibiting a *p*-value between 0.05 and 0.0056 were thought to be suggesting of a possible association. LDSC packages (32) in R version 4.0.2 were used for all analyses.

### MR estimates

Causal estimates for the impact of genetically predicted serum Ca, VD, and VK levels on outcome variables were assessed with an inverse-variance-weighting (IVW) approach using a fixed-effects model. Weighted median, MR-Egger regression, and MR Pleiotropy RESidual Sum and Outlier (MR-PRESSO) strategies were also employed to improve the reliability and robustness of study conclusions. The weighted median approach assumes that a minimum of 50% of available information is based upon valid Ivs (32). The MR-Egger approach offers validity despite permitting the presence of invalid variants, but can yield wide confidence interval (CI) values (33). The MR-PRESSO approach enables researchers to detect and correct for any analyses detected through IVW linear regression analyses (34). Bonferroni correction of these results was used as above, with a *p*-value < 0.0056 as the significance threshold and a *p*-value between 0.05 and 0.0056 being indicative of a possible association. Odds ratios (ORs) are given for every 1 standard deviation (SD) difference in serum levels of Ca, VD, and VK. The TwoSampleMR (35) and MRPRESSO (36) packages in R version 4.0.2 was used for all analyses.

### Analyses of pleiotropy and heterogeneity

Analyzing potential pleiotropy is vital given that pleiotropic Ivs have the potential to have an indirect impact on study outcomes, serving as confounders of MR analysis efforts. A range of strategies were utilized herein in an effort to detect possible pleiotropy. Initially, heterogeneity among Ivs when utilizing the fixed-effects IVW approach was detected through Cochrane’s *Q* test. Lower levels of heterogeneity are indicative of the possibility that estimates between Ivs vary based on random chance, which can only occur when pleiotropic effects are not evident. In cases where significant heterogeneity was detected, a multiplicative random-effects IVW model would be implemented. An MR-Egger intercept was additionally performed, with a zero intercept (*p* > 0.05) being indicative of a lack of any pleiotropic bias. The MR-PRESSO method was additionally used for global heterogeneity testing and to detect horizontal pleiotropy (36). To assess the extent to which these associations were under the influence of any one SNP, a leave-one-out sensitivity analysis was conducted. Moreover, all SNPs included in the GWAS catalog database (37) were searched, with the goal of determining the association between those SNPs and risk factors pertaining to CVD incidence and longevity outcomes. After removing pleiotropic SNPs, causal associations were also analyzed.

### RCS analysis

Restrictive cubic spline analyses entail the use of a piecewise polynomial function capable of examining non-linear relationships between predictors and outcomes in a flexible manner (38). Here, spline models were adjusted for covariates including age, sex, body mass index (BMI), genotype batch, assessment center, and Townsend deprivation index (TDI) (39). Multivariate logistic analyses were used to examine relationships between serum Ca or VD concentrations and CVD incidence at the 25, 50, 75, and 95th centiles. When less than 20% of covariate data was absent, these missing values were accounted for through multiple imputations based upon five replicates and a chained equation method using the R MI procedure. Baseline categorical data were summarized across serum Ca and VD concentrations as percentages, while continuous variables were summarized using means and SDs. A two-sided *p* < 0.05 was the threshold of significance. R version 4.0.2 was used for all analyses.

## Results

### Genetic correlations between serum Ca, vitamin D, vitamin K, and CVDs

**FIGURE 1:**
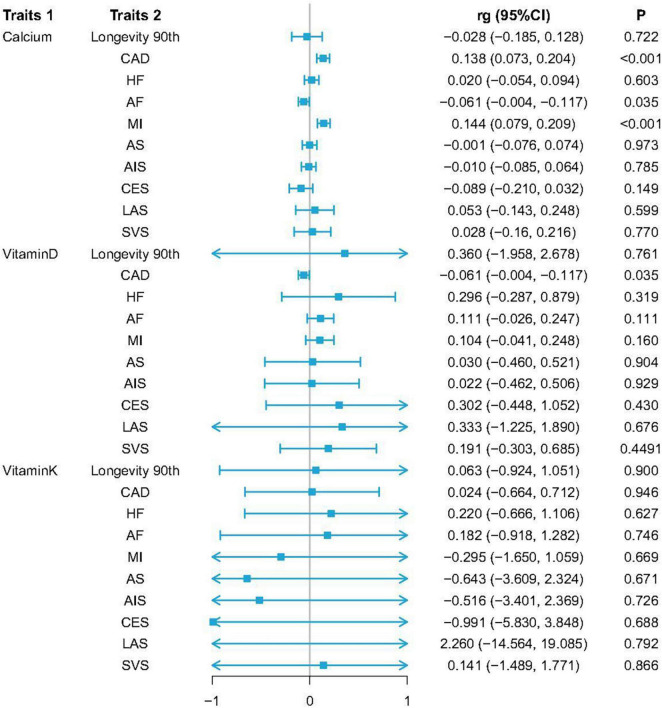
Genetic correlation estimates for the associations between serum levels of calcium (Ca), vitamin D (VD), and vitamin K1 (VK1), and cardiovascular diseases (CVDs) as well as longevity. Traits 1 and 2 respectively, correspond to study exposures and outcomes. Error bars denote 95% confidence interval (CIs).

When examining genetic correlations pertaining to serum Ca levels, the positive genetic correlation following Bonferroni correction was detected for MI [*r*_g_ (SE) = 0.890 (0.012); *p* = 1.356E–04] and CAD [*r*_g_ (SE) = 0.868 (0.014); *p* = 3.601E–04]. Serum Ca levels also exhibited a negative genetic correlation with AF, much as VD levels did with CAD [*r*_g_ (SE) = −0.061 (0.029); *p* = 0.035; *p*_adj_ = 0.350] (Figure 1 and Supplementary Table 1). No genetic correlations were detected when examining the relationship between VK (VK1, circulating phylloquinone concentrations) and any CVD subtypes. Results from SNV-based heritability testing suggest that these three tested exposures were unrelated to longevity (*p*_range_ = 0.722–0.900).

### Causal associations between serum Ca, vitamin D, vitamin K, and CVDs

**FIGURE 2:**
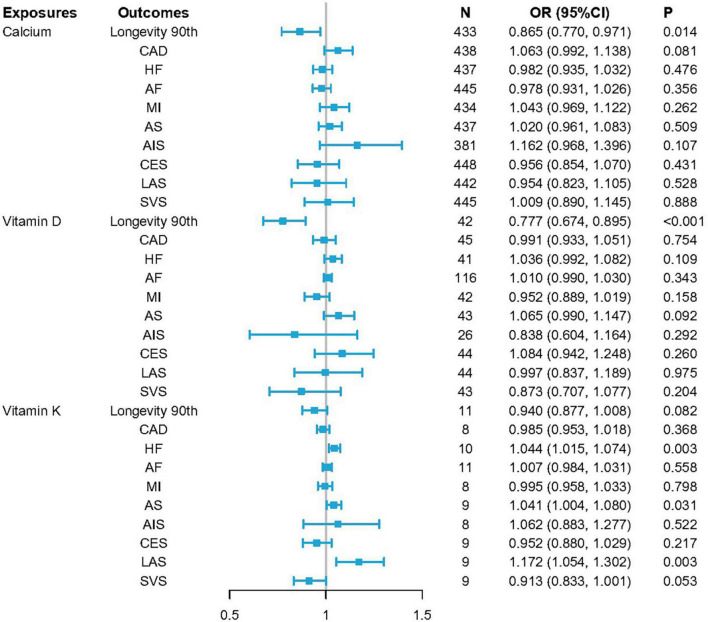
OR_SD_ for causal associations between serum levels of calcium (Ca), vitamin D (VD), and vitamin K1 (VK1), and cardiovascular diseases (CVDs) as well as longevity. Odds ratio (OR) estimates for individual single nucleotide variants (SNVs) were made using the inverse-variance-weighted (IVW) method. OR_SD_ = OR for standard deviation (SD) unit increases in risk factors.

When conducting IVW MR analyses, higher VK1 levels were found to be strongly related to the risk of HF (*p* = 0.003, OR per 1-SD increase: 1.044; CI: 1.015–1.074) and LAS (*p* = 0.003, OR per 1-SD increase: 1.172; CI: 1.054–1.302). Potential associations were also observed between increases in VK1 levels and the risk of AS (*p* = 0.031, OR per 1-SD increase: 1.041; CI: 1.004–1.080). MR analyses also indicated a suggestive association between higher serum Ca concentrations and reduced odds of longevity (*p* = 0.014, OR per 1-SD increase: 0.865; CI: 0.770–0.971), with VD levels being significantly associated with reduced odds of longevity (*p* = 4.620E–04, OR per 1-SD increase: 0.777; CI: 0.674–0.895) (Figure 2 and Supplementary Table 3). Fixed-effects IVW estimates failed to reveal any causal associations between VK levels and longevity outcomes. Serum Ca or VD levels were also not found to be causally related to all tested CVDs.

To ensure that the causal inferences drawn from MR analyses are valid, it is critical that it be established that SNV-outcome relationships are the result of a given exposure and not the consequence of horizontal pleiotropy or a similar mechanism. Pleiotropy-resistant sensitivity analyses including Cochrane’s *Q* test, as well as MR-Egger intercept, MR-PRESSO, and leave-one-out sensitivity analyses were thus performed (Supplementary Figures 2, 3). Observed relationships between VK levels and HF/LAS/AS remained robust in these analyses (Cochrane’s *Q*_HF_ = 13.088, *p*_HF_ = 0.159; MR-Egger intercept, *p*_HF_ = 0.197; and MR-PRESSO, *p*_HF_ = 0.181; Cochrane’s *Q*_LAS_ = 3.631, *p*_LAS_ = 0.889; MR-Egger intercept, *p*_LAS_ = 0.010; and MR-PRESSO, *p*_LAS_ = 0.900; and Cochrane’s *Q*_AS_ = 4.678, *p*_AS_ = 0.791; MR-Egger intercept, *p*_AS_ = 0.953; and MR-PRESSO, *p*_AS_ = 0.804) (Table 2 and Supplementary Table 4). Consistent directionality was also evident for the relationships between increases in serum Ca/VD levels and longevity across all of these sensitivity analyses (Cochrane’s *Q*_calcium_ = 372.154, *p*_calcium_ = 0.983; MR-Egger intercept, *p*_calcium_ = 0.197; and MR-PRESSO, *p*_calcium_ = 0.983 and Cochrane’s *Q*_vitamin D_ = 26.940, *p*_vitamin D_ = 0.956; MR-Egger intercept, *p*_vitamin D_ = 0.339; and MR-PRESSO, *p*_vitamin D_ = 0.961) (Table 2 and Supplementary Table 4).

**TABLE 2:** Results of potential pleiotropy and heterogeneity assessments.

Exposures	Outcomes	Cochran’s *Q* statistic	*P*-value for Cochran’s *Q*	*p*-value for intercept	MR-PRESSO global test
Calcium	CAD	665.368	1.015E–11	0.789	<1E–04
	MI	606.473	6.667E–08	0.758	<1E–04
	AF	685.478	1.311E–12	0.891	<1E–04
	HF	505.539	0.012	**0.020**	**0.011**
	AIS	358.083	0.784	0.865	0.787
	AS	518.219	0.004	0.821	**0.004**
	CES	461.504	0.308	0.912	0.317
	LAS	433.451	0.592	0.910	0.593
	SVS	486.558	0.080	0.806	0.084
	Longevity 90	372.154	0.983	0.197	0.983
Vitamin D	CAD	31.876	0.913	0.709	0.919
	MI	25.366	0.974	0.394	0.977
	AF	102.814	0.785	0.286	0.790
	HF	42.513	0.363	0.600	0.419
	AIS	13.268	0.973	0.885	0.973
	AS	22.313	0.995	**0.030**	0.995
	CES	40.566	0.577	0.582	0.599
	LAS	48.618	0.257	0.879	0.274
	SVS	69.788	0.005	0.183	**0.004**
	Longevity 90	26.940	0.956	0.339	0.961
Vitamin K1	CAD	1.659	0.976	0.309	0.979
	MI	5.118	0.646	0.838	0.662
	AF	0.969	1.000	0.532	1.000
	HF	13.088	0.159	0.197	0.181
	AIS	14.952	0.037	**0.024**	**0.046**
	AS	4.678	0.791	0.953	0.804
	CES	7.650	0.468	0.125	0.489
	LAS	3.631	0.889	**0.010**	0.900
	SVS	4.978	0.760	0.112	0.756
	Longevity 90	8.363	0.593	0.421	0.617

*P*-values below the threshold of 0.05 are displayed in bold.

### Plateau points for serum Ca-associated exposures

**FIGURE 3:**
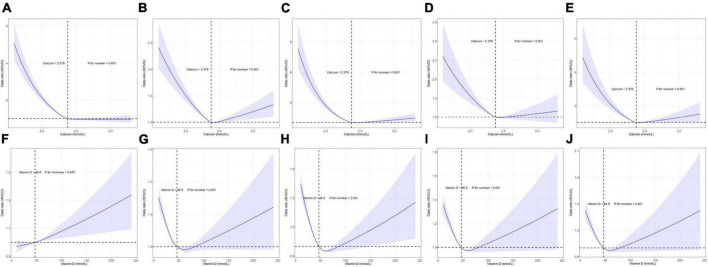
Restricted cubic spline model-based analyses of the association between serum calcium (Ca)/vitamin D (VD) levels and the risk of cardiovascular diseases (CVDs). **(A–E)** Curvilinear relationships between serum Ca levels and atrial fibrillation (AF), coronary artery disease (CAD), heart failure (HF), myocardial infarction (MI), and stroke. **(F–J)** Curvilinear relationships between VD concentrations and AF, CAD, HF, MI, and stroke. The *y*-axis represents the log of logistic regression model-derived odds ratios (ORs), while the shaded area denotes the corresponding 95% confidence interval (CIs) for these adjusted ORs. A plateau in CVD risk was evident in the risk function.

In total, serum Ca and VD level data in the UK Biobank were available for 429,863 and 448,777 individuals, respectively. Mean ages and gender distributions for these individuals are summarized in Supplementary Table 5. Individual VK data was not available with respect to CVD incidence. Through the use of an RCS regression analysis, curvilinear associations were detected between CVDs and both serum Ca and VD levels. All CVDs (CAD, AF, HF, MI, stroke) at the serum Ca plateau point (2.376 mmol/L, *p* < 0.001) were significantly different, whereas four CVDs (MI, stroke, HF, CAD) differed significantly at the VD plateau point (46.8 nmol/L, *p* < 0.001) (Figure 3).

## Discussion

In this study, SNV-based genetic correlations and potential causal relationships between levels of serum Ca-associated exposures and both CVD and longevity were assessed. The resultant data were complementary, indicating that both serum Ca and VD were genetically but not causally related to CVD incidence, whereas these serum Ca and VD levels were causally but not genetically associated with longevity. VK levels were also causally related to CVD incidence, but not related to CVDs or longevity in genetic correlation analyses. Genetic correlation tests heritability and co-heritability between two traits, while MR analysis assesses possible causality between exposures and outcomes, which complement each other to indicate a possible relationship between two traits. Plateau points for serum Ca and VD levels associated with reduced CVD risk were also analyzed. Multivariate analyses ultimately revealed that serum Ca and VD levels were non-linearly related to CVD incidence after adjusting for confounding factors (*p* < 0.001) (Figure 3). In this European population, the serum Ca plateau point was 2.376 mmol/L, indicating that this concentration was associated with the minimum CVD incidence, in addition to falling within the standard the normal serum Ca clinical reference range (2.2–2.6 mmol/L) (40). Moreover, a VD concentration of 46.8 nmol/L was associated with the lowest risk of CVD incidence.

### Serum Ca and CVD

Calcium is a divalent cation that plays essential roles in diverse physiological processes such as nerve excitation, muscle contraction, the mineralization of the skeleton, and coagulatory function (41, 42). In observational analyses, serum Ca concentrations have been shown to be positively correlated with CVD risk (43, 44). RCT-derived evidence suggests that Ca supplementation, which can lead to acute or persistently elevated serum Ca concentrations (45, 46), can result in a modest increase in the risk of MI and other cardiovascular events (47). An MR analysis of 184,305 participants (including 60,801 CAD cases, of which ∼70% had experienced MI, and 123,504 non-cases) revealed an association between a genetic predisposition toward elevated serum Ca levels and a higher risk of MI and CAD (48). LDSC analyses in this study confirmed a significant positive genetic relationship between serum Ca levels and both CAD (*p* = 3.601E–04) and MI (*p* = 1.356E–04). Insufficient evidence is currently available regarding the association between serum Ca and AF incidence, in line with the weak negative genetic correlation between these variables observed in this study. The inverse genetic associations between serum Ca and both CAD and MI, and serum Ca and AF may be due to the non-linear relationship between Ca and CVDs itself. Both genetic correlation analysis and MR are based on the assumption of linear relationship between serum Ca and CVDs, which may need to be further explained by observational data. However, our results of RCS just confirmed that there is a U-shaped relationship between them through individual observational data analysis.

Recent epidemiological evidence further suggests that circulating Ca levels are associated with CVD-related mortality and with longevity (43, 44, 49, 50). Specifically, elevated levels of serum Ca were linked to an increase in the odds of non-fatal CVD (HR = 1.12, 95% CI 1.10–1.14, MI: 1.19, 1.14–1.25) and fatal CVD (HR = 1.41, 95% CI 1.35–1.47; MI: 1.41, 1.31–1.51) (44). The MR analysis conducted herein also revealed a causal relationship between serum Ca levels and longevity (*p* = 0.014). Higher serum Ca levels were significantly associated with longevity and were negatively correlated with the incidence of AS, SVS, AIS, MI, and CAD, although these latter relationships were not significant. The combination of MR and LDSC results revealed a correlation between serum Ca levels, CVDs, and longevity.

### Vitamin D and CVD

Vitamin D plays an essential role in regulating Ca homeostasis, but the expression of nuclear VD receptor (VDR) by cardiomyocytes and vascular endothelial cells suggests that VD may be directly involved in the development and progression of CVD (51). These data thus prompted a more in-depth analysis of VD in addition to Ca.

In published studies, a 1.41-fold greater risk of CVD mortality (95% CI: 1.18–1.68) for individuals in the lowest plasma VD quintile based on a meta-analysis of prospective cohort studies. Acute VD deficiencies can contribute to inflammation and impaired insulin secretion, thereby increasing the odds of plaque rupture and arterial thrombosis. Chronically insufficient VD levels can contribute to increased arterial stiffness (52). Overall, VD deficiencies are detrimental to cardiovascular or longevity outcomes over any time scale. Observational results suggest that low levels of serum 25-hydroxyVD [25(OH)D], with is the primary form in which VD is stored, are related to an elevated risk of CVD incidence and mortality (53). VD deficiency has also been found to be associated with a more severe cardiovascular risk profile and increased CAD prevalence (54). VD was also shown to suppress NF-κB pathway signaling within cells to inhibit the progression of CAD, highlighting a possible mechanism whereby VD may mitigate vascular inflammation and atherosclerosis (55). The genetic and causal association analyses conducted herein revealed VD levels to be genetically related to CAD (*p* = 0.035) and causally related to longevity (*p* = 4.620E–04), confirming the association between VD exposure and these endpoints. An inverse relationship was observed between VD and CES, AS, HF, and AF incidence, but these relationships did not attain the level of statistical significance.

### Vitamin K and CVD

As a fat-soluble vitamin, VK is required for the activation of certain proteins and has been suggested to play some role in CVD incidence. Through anti-inflammatory activity that has been observed *in vitro* and *in vivo*, VK can potentially protect against vascular calcification, thus lowering the odds of CVD development and all-cause mortality (17). One observational prospective analysis of 601 individuals found lower VD and VK levels to be related to adverse cardiac remodeling and greater all-cause mortality risk (56). Conversely, a meta-analysis of three cohorts in the USA found VK1 levels to be related to all-cause mortality risk but unrelated to CVD (57). Circulating VK1 levels were also found not to be causally associated with CHD in a prior two-sample MR study (RR = 1.00, 95% CI: 0.98–1.04) (58). Here, analyses of different CVD subtypes revealed VK1 levels to be causally associated with HF (*p* = 0.003), AS (*p* = 0.031), and LAS (*p* = 0.003). However, epidemiological data pertaining to correlations between VK1 and various CVD subtypes are lacking at present, underscoring the need for further research focused on this topic and the underlying mechanisms that link VK levels between CVD or other health outcomes.

### Clinical implications

Calcium supplementation is a common practice in the USA, and there is rising clinical interest with respect to the association between these supplements and CVD. Some work suggests that Ca supplements may lower blood pressure and contribute to better serum lipid profiles, yet they also have the potential to increase serum Ca levels, thereby elevating the risk of vascular calcification and concomitant CVD event incidence. Perhaps unsurprisingly, prior research has yielded conflicting results with respect to the relationships between CVD and Ca supplementation (13). This issue is made more complex by the fact that many adults seek to improve their bone health through the combined intake of Ca and VD supplements despite the inconclusive evidence suggesting possible relationship between Ca intake and the risk of CVD (53). Some adverse effects have been reported in individuals utilizing supplemental VD and Ca alone or in combination, with these effects likely being attributable to the dose of supplemental Ca utilized (59). The present results suggest a genetic relationship between serum Ca, VD concentrations, and CVD incidence such that these serum biomarkers may offer value for the selection of appropriate nutritional interventions designed to mitigate CVD-related risk. Importantly, this study enabled the establishment of threshold Ca and VD concentrations in CVD patients and healthy controls, revealing that serum Ca and VD levels of 2.376 mmol/L and 46.8 nmol/L, respectively, were related to the lowest risk of CVD development among individuals of European heritage. When these levels fall too far above or below these levels, they may contribute to CVD development. A study of 441,738 individuals during a median follow-up time of 21 years found that serum Ca concentrations greater than 2.40 nmol/L were associated with increased risk of non-fatal CVD (44). Another study discovered L-shaped associations between VD level and CVD mortality. When VD concentrations were less than 27.70 nmol/L, the risk of death from CVD was increased. When VD concentrations exceeded 54.40 nmol/L, there was no association with all-cause mortality in America (60). Although no studies have yet provided exact epidemiological data on serum Ca and VD concentrations, the range of concentrations given by previous studies supports our results. At the same time, the Ca (2.376 mmol/L) and VD (46.8 nmol/L) plateau points of CVD are within the standard the normal clinical reference range. Accordingly, the intake of Ca or VD from primarily dietary sources may be most appropriate, reserving the minimum necessary Ca/VD supplementation for individuals dealing with Ca/VD intake deficiencies following the exhaustion of other forms of dietary modification (53).

When analyzing supplemental VK1 intake, following adjustment for confounding lifestyle and demographic factors, moderate-to-high VK1 intake levels (87–192 μg/days) were related to a decrease in the odds of all-cause [HR (95% CI): 0.76 (0.72, 0.79)], and CVD-related [HR (95% CI): 0.72 (0.66, 0.79)] (61). These findings confirmed that VK1 levels were related to AS, LAS, and HF. However, individual VK1 data were unavailable such that it was not possible to estimate the threshold levels necessary to minimize the risk of CVD development. As such, further research will be needed to provide specific guidance regarding supplemental VK1 dosing in different populations.

### Strengths and limitations

A major strength of this study is that the analyses of serum Ca-associated exposures for the nine included CVD types and overall longevity were performed using the largest GWAS datasets available. Causal inferences should generally be based upon several study types given that MR analyses are based on three major assumptions that are not always met or fully testable (62, 63). Genetic correlation analyses were thus used herein in an effort to complement MR-related research design limitations. Furthermore, these outcomes included both specific analyses for nine CVD subtypes as well as longevity as a control outcome for co-analyses, thereby strengthening the overall reliability of these findings. An additional strength of this approach is that the genetic instruments employed herein were selected based on a recent European population GWAS dataset for individuals with accessible serum Ca and plasma VD/K1 levels together with summary-level information regarding CVDs and longevity. These results are not likely to have been affected by population stratification bias given that these were GWAS data for individuals who were primarily of European ancestry. Lastly, correlation analyses of the associations between serum Ca and VD levels and the CVD risk factors enabled the estimation of the serum Ca and VD levels associated with the minimum CVD risk, thus enabling the establishment of recommended threshold levels for these nutrients aimed at mitigating the odds of CVD development.

There are certain limitations to this analysis. For one, the GWAS study used for these analyses was derived from a public database pertaining to a European population, and the results may thus not be applicable to populations of Asian, African, or other ancestries. In addition, individual-level VK data were not available for these European CVD patients, precluding the establishment of optimal concentrations of this vitamin for cardiovascular health.

## Conclusion

Our findings support a genetic correlation between serum Ca and VD and CVD risk, and a causal relationship between VK1 levels and CVD risk. The optimal serum Ca and vitamin plasma D concentrations associated with the minimum risk of CVDs were 2.376 mmol/L and 46.8 nmol/L, respectively. Whether plasma VK1 levels can contribute to improved CVD outcomes and extend lifespan, however, has yet to be established.

## Data availability statement

Publicly available datasets were analyzed in this study. These data can be found here: Summary statistics from the GWAS used in this study are publicly accessible in the published literature and UK Biobank Resource which are shown in Table 1.

## Author contributions

XN, LL, YY, CZ, and HS conceived and designed the study, literature search, and wrote the original draft. YY, YL, RL, ZC, and WH did the data collection, formal analysis, and methodology. XN, LL, HS, and QZ did the visualization and methodology. LS, XZ, ZY, HZ, and SZ accessed and verified the data. CH, ZY, and HY did project administration and coordination and reviewed and edited the manuscript. All authors had final responsibility for the decision to submit for publication.
