# Supplementary material for: The genetic correlation and causal association between key factors that influence vascular calcification and cardiovascular disease incidence
Source: Front Cardiovasc Med. 2023 Jan 26;10:1096662. doi: 10.3389/fcvm.2023.1096662 (PMC9908996; doi:10.3389/fcvm.2023.1096662)
Supplement: Supplementary file 1 [file Data_Sheet_1.docx]

**Supplementary Figure**

**Supplementary Figure 1: The flowchart of this study.**

**Supplementary Figure 2: Funnel plots corresponding to causal estimate (β_IV_) and instrument strength (1/SE_IV_) for analyzed variants as instrumental variables for outcomes.** Causal estimate calculates were based on the log of the odds ratio per genetically predicted standard deviation unit increase in the risk factor. Red lines denote causal effects based on the use of an inverse-variance weighted (IVW) model, with the null hypothesis being represented with dotted lines.

**Supplementary Figure 3: Leave-one-out Histogram for the MR analysis of the associations between serum levels of Ca, vitamin D, and vitamin K1, and CVDs as well as longevity.**

| **Supplementary table 1. Genetic Correlation Estimates from LDSC Regression** | | | | | | | | | | |  |
| --- | --- | --- | --- | --- | --- | --- | --- | --- | --- | --- | --- |
| **Phenotype 1** | **Phenotype 2** | **N_snp_** | **rg** | **se** | **rg intercept (SE)** | ***p*-values** | | ***p*_adj_** | **rg intercept (SE)** | | |
| Calcium | CAD | 1022559 | 0.138 | 0.034 | 0.138 (0.034) | **3.601E-05** | 3.601E-04 | | | 0.868 (0.014) |  |
|  | MI | 1022070 | 0.144 | 0.033 | 0.144 (0.033) | **1.356E-05** | 1.356E-04 | | | 0.890 (0.012) |  |
|  | AF | 1022780 | -0.061 | 0.029 | -0.061 (0.029) | **0.035** | 0.350 | | | 0.991 (0.015) |  |
|  | HF | 1019924 | 0.020 | 0.038 | 0.020 (0.038) | 0.603 | 1.000 | | | 1.010 (0.010) |  |
|  | AIS | 1021985 | -0.010 | 0.038 | -0.010 (0.038) | 0.785 | 1.000 | | | 1.004 (0.011) |  |
|  | AS | 1021802 | -0.001 | 0.038 | -0.001 (0.038) | 0.973 | 1.000 | | | 1.022 (0.011) |  |
|  | CES | 1021687 | -0.089 | 0.062 | -0.089 (0.062) | 0.149 | 1.000 | | | 1.054 (0.011) |  |
|  | LAS | 1022310 | 0.053 | 0.100 | 0.053 (0.1) | 0.599 | 1.000 | | | 1.089 (0.010) |  |
|  | SVS | 1021837 | 0.028 | 0.096 | 0.028 (0.096) | 0.770 | 1.000 | | | 1.067 (0.010) |  |
|  | Longevity 90th | 1020742 | -0.028 | 0.080 | -0.028 (0.08) | 0.722 | 1.000 | | | 1.037 (0.020) |  |
| Vitamin D | CAD | 1022559 | -0.061 | 0.029 | -0.061 (0.029) | **0.035** | 0.350 | | | 0.991 (0.015) |  |
|  | MI | 1022070 | 0.104 | 0.074 | 0.104 (0.074) | 0.160 | 1.000 | | | 0.897 (0.008) |  |
|  | AF | 1029530 | 0.111 | 0.069 | 0.111 (0.069) | 0.111 | 1.000 | | | 0.881 (0.008) |  |
|  | HF | 1019924 | 0.296 | 0.297 | 0.296 (0.297) | 0.319 | 1.000 | | | 1.000 (0.009) |  |
|  | AIS | 1021985 | 0.022 | 0.247 | 0.022 (0.247) | 0.929 | 1.000 | | | 1.029 (0.035) |  |
|  | AS | 1021802 | 0.030 | 0.250 | 0.030 (0.25) | 0.904 | 1.000 | | | 1.033 (0.009) |  |
|  | CES | 1021687 | 0.302 | 0.383 | 0.302 (0.383) | 0.430 | 1.000 | | | 0.537 (0.116) |  |
|  | LAS | 1022310 | 0.333 | 0.795 | 0.333 (0.795) | 0.676 | 1.000 | | | 0.698 (0.140) |  |
|  | SVS | 1021837 | 0.191 | 0.252 | 0.191 (0.252) | 0.449 | 1.000 | | | 1.074 (0.007) |  |
|  | Longevity 90th | 1020742 | 0.360 | 1.183 | 0.360 (1.183) | 0.761 | 1.000 | | | 1.023 (0.009) |  |
| Vitamin K1 | CAD | 969402 | 0.024 | 0.351 | 0.024 (0.351) | 0.946 | 1.000 | | | 0.877 (0.009) |  |
|  | MI | 968913 | -0.295 | 0.691 | -0.295 (0.691) | 0.669 | 1.000 | | | 0.894 (0.008) |  |
|  | AF | 969623 | 0.182 | 0.561 | 0.182 (0.561) | 0.746 | 1.000 | | | 1.040 (0.017) |  |
|  | HF | 966767 | 0.220 | 0.452 | 0.220 (0.452) | 0.627 | 1.000 | | | 1.007 (0.008) |  |
|  | AIS | 968828 | -0.516 | 1.472 | -0.516 (1.472) | 0.726 | 1.000 | | | 1.026 (0.007) |  |
|  | AS | 968645 | -0.643 | 1.514 | -0.643 (1.514) | 0.671 | 1.000 | | | 1.037 (0.007) |  |
|  | CES | 968530 | -0.991 | 2.469 | -0.991 (2.469) | 0.688 | 1.000 | | | 1.057 (0.007) |  |
|  | LAS | 969153 | 2.260 | 8.584 | 2.260 (8.584) | 0.792 | 1.000 | | | 1.105 (0.007) |  |
|  | SVS | 968680 | 0.141 | 0.832 | 0.141 (0.832) | 0.866 | 1.000 | | | 1.069 (0.007) |  |
|  | Longevity 90th | 967585 | 0.063 | 0.504 | 0.063 (0.504) | 0.900 | 1.000 | | | 1.006 (0.008) |  |

Note: *p*-values below the threshold of 0.05 are displayed in bold.

*p*_adj_ were adjusted by Bonferroni correction for each phenotype.

| **Supplementary table 2. Causal estimates from each MR approach and MR Heterogeneity Estimates for each exposure and outcome risk (p< 5E-08)** | | | | | | |
| --- | --- | --- | --- | --- | --- | --- |
|  |  |  | **IVW** | | | |
| **Exposures** | **Outcomes** | **N_snp_** | **BETA** | **SE** | **OR(95%CI)** | ***p*-value** |
| Calcium | CAD | 219 | 0.067 | 0.033 | 1.070 (1.003, 1.141) | 0.042 |
|  | MI | 220 | 0.053 | 0.036 | 1.055 (0.982, 1.132) | 0.143 |
|  | AF | 225 | -0.033 | 0.022 | 0.968 (0.927, 1.011) | 0.143 |
|  | HF | 221 | -0.015 | 0.027 | 0.985 (0.934, 1.038) | 0.568 |
|  | AIS | 221 | -0.015 | 0.027 | 0.985 (0.934, 1.038) | 0.568 |
|  | AS | 228 | -0.015 | 0.031 | 0.985 (0.927, 1.046) | 0.617 |
|  | CES | 230 | 0.004 | 0.065 | 1.004 (0.884, 1.14) | 0.956 |
|  | LAS | 226 | -0.040 | 0.085 | 0.961 (0.813, 1.135) | 0.638 |
|  | SVS | 226 | 0.029 | 0.072 | 1.030 (0.895, 1.185) | 0.684 |
|  | Longevity90 | 224 | -0.235 | 0.060 | 0.790 (0.702, 0.889) | 9.223E-05 |
| Vitamin D | CAD | 12 | -0.041 | 0.051 | 0.959 (0.868, 1.061) | 0.418 |
|  | MI | 12 | -0.059 | 0.056 | 0.943 (0.844, 1.053) | 0.294 |
|  | AF | 21 | 0.013 | 0.022 | 1.013 (0.970, 1.058) | 0.568 |
|  | HF | 13 | 0.034 | 0.025 | 1.034 (0.985, 1.086) | 0.172 |
|  | AIS | 13 | 0.034 | 0.025 | 1.034 (0.985, 1.086) | 0.172 |
|  | AS | 12 | 0.047 | 0.049 | 1.048 (0.952, 1.154) | 0.336 |
|  | CES | 13 | 0.065 | 0.064 | 1.067 (0.941, 1.209) | 0.311 |
|  | LAS | 14 | -0.043 | 0.081 | 0.958 (0.818, 1.122) | 0.592 |
|  | SVS | 13 | -0.238 | 0.101 | 0.789 (0.647, 0.961) | 0.018 |
|  | Longevity90 | 13 | -0.164 | 0.091 | 0.849 (0.710, 1.014) | 0.071 |

**Supplementary table 3. Causal estimates from each MR approach and MR Heterogeneity Estimates for each exposure and outcome risk (p< 1E-05)**

|  |  |  | **IVW** | | | | **Weighted Median** | | | | **Weighed Mode** | | | | **MR-PRESSO** | | | |
| --- | --- | --- | --- | --- | --- | --- | --- | --- | --- | --- | --- | --- | --- | --- | --- | --- | --- | --- |
| **Exposures** | **Outcomes** | **N_snp_** | **BETA** | **SE** | **OR(95%CI)** | ***p*-value** | **BETA** | **SE** | **OR(95%CI)** | ***p*-value** | **BETA** | **SE** | **OR(95%CI)** | ***p*-value** | **MR-PRESSO** | **SE** | **OR(95%CI)** | ***p*-value** |
| Calcium | CAD | 438 | 0.061 | 0.035 | 1.063 (1.005, 1.123) | 0.081 | -0.022 | 0.081 | 0.978 (0.835, 1.146) | 0.784 | 0.016 | 0.047 | 1.017 (0.927, 1.114) | 0.726 | 0.061 | 0.035 | 1.063 (0.992, 1.138) | 0.082 |
|  | MI | 434 | 0.042 | 0.037 | 1.043 (0.980, 1.109) | 0.262 | 0.000 | 0.093 | 1.000 (0.833, 1.201) | 1.000 | -0.012 | 0.051 | 0.988 (0.893, 1.093) | 0.816 | 0.042 | 0.037 | 1.043 (0.969, 1.122) | 0.263 |
|  | AF | 445 | -0.023 | 0.025 | 0.978 (0.940, 1.016) | 0.356 | 0.018 | 0.067 | 1.018 (0.893, 1.161) | 0.786 | 0.004 | 0.033 | 1.004 (0.942, 1.071) | 0.898 | -0.023 | 0.025 | 0.978 (0.931, 1.026) | 0.357 |
|  | HF | 437 | -0.018 | 0.025 | 0.982 (0.938, 1.028) | 0.476 | 0.026 | 0.061 | 1.026 (0.910, 1.157) | 0.674 | 0.003 | 0.037 | 1.003 (0.932, 1.079) | 0.945 | -0.018 | 0.025 | 0.982 (0.935, 1.032) | 0.476 |
|  | AIS | 381 | 0.150 | 0.093 | 1.162 (0.968, 1.396) | 0.107 | 0.598 | 0.259 | 1.818 (1.094, 3.021) | **0.021** | 0.278 | 0.143 | 1.320 (0.998, 1.747) | 0.052 | 0.150 | 0.091 | 1.162 (0.973, 1.388) | 0.098 |
|  | AS | 437 | 0.020 | 0.030 | 1.020 (0.966, 1.077) | 0.509 | -0.003 | 0.057 | 0.997 (0.892, 1.114) | 0.953 | 0.023 | 0.044 | 1.023 (0.938, 1.116) | 0.607 | 0.020 | 0.030 | 1.020 (0.961, 1.083) | 0.509 |
|  | CES | 448 | -0.045 | 0.057 | 0.956 (0.854, 1.070) | 0.431 | -0.012 | 0.123 | 0.988 (0.776, 1.259) | 0.925 | -0.046 | 0.090 | 0.955 (0.800, 1.140) | 0.613 | -0.045 | 0.058 | 0.956 (0.853, 1.072) | 0.439 |
|  | LAS | 442 | -0.048 | 0.075 | 0.954 (0.823, 1.105) | 0.528 | -0.016 | 0.183 | 0.984 (0.687, 1.409) | 0.929 | 0.028 | 0.117 | 1.028 (0.818, 1.292) | 0.811 | -0.048 | 0.075 | 0.954 (0.824, 1.104) | 0.525 |
|  | SVS | 445 | 0.009 | 0.064 | 1.009 (0.890, 1.145) | 0.888 | 0.020 | 0.137 | 1.021 (0.781, 1.334) | 0.881 | -0.019 | 0.146 | 0.981 (0.737, 1.307) | 0.898 | 0.009 | 0.067 | 1.009 (0.884, 1.151) | 0.893 |
|  | Longevity90 | 433 | -0.145 | 0.059 | 0.865 (0.770, 0.971) | **0.014** | -0.368 | 0.163 | 0.692 (0.502, 0.953) | **0.024** | -0.212 | 0.092 | 0.809 (0.676, 0.968) | **0.021** | -0.145 | 0.055 | 0.865 (0.777, 0.963) | 0.008 |
| Vitamin D | CAD | 45 | -0.009 | 0.030 | 0.991 (0.933, 1.051) | 0.754 | -0.030 | 0.047 | 0.971 (0.885, 1.065) | 0.533 | -0.012 | 0.048 | 0.988 (0.899, 1.086) | 0.798 | -0.009 | 0.026 | 0.991 (0.942, 1.042) | 0.715 |
|  | MI | 42 | -0.049 | 0.035 | 0.952 (0.889, 1.019) | 0.158 | -0.049 | 0.058 | 0.952 (0.849, 1.068) | 0.402 | -0.020 | 0.053 | 0.980 (0.883, 1.087) | 0.698 | -0.049 | 0.027 | 0.952 (0.902, 1.005) | 0.080 |
|  | AF | 116 | 0.010 | 0.010 | 1.010 (0.990, 1.030) | 0.343 | 0.016 | 0.021 | 1.016 (0.976, 1.057) | 0.448 | 0.022 | 0.015 | 1.022 (0.993, 1.053) | 0.143 | 0.010 | 0.010 | 1.010 (0.991, 1.029) | 0.318 |
|  | HF | 41 | 0.035 | 0.022 | 1.036 (0.992, 1.082) | 0.109 | 0.032 | 0.028 | 1.033 (0.978, 1.090) | 0.248 | 0.035 | 0.032 | 1.035 (0.973, 1.102) | 0.273 | 0.035 | 0.023 | 1.036 (0.991, 1.083) | 0.128 |
|  | AIS | 26 | -0.176 | 0.167 | 0.838 (0.604, 1.164) | 0.292 | -0.415 | 0.317 | 0.660 (0.355, 1.229) | 0.191 | -0.345 | 0.229 | 0.708 (0.452, 1.109) | 0.131 | -0.176 | 0.122 | 0.838 (0.660, 1.064) | 0.160 |
|  | AS | 43 | 0.063 | 0.038 | 1.065 (0.990, 1.147) | 0.092 | 0.116 | 0.101 | 1.123 (0.920, 1.370) | 0.254 | 0.065 | 0.053 | 1.067 (0.961, 1.185) | 0.222 | 0.063 | 0.027 | 1.065 (1.010, 1.124) | 0.026 |
|  | CES | 44 | 0.081 | 0.072 | 1.084 (0.942, 1.248) | 0.260 | 0.021 | 0.108 | 1.021 (0.826, 1.261) | 0.848 | 0.039 | 0.112 | 1.040 (0.834, 1.296) | 0.729 | 0.081 | 0.070 | 1.084 (0.946, 1.243) | 0.252 |
|  | LAS | 44 | -0.003 | 0.090 | 0.997 (0.837, 1.189) | 0.975 | 0.025 | 0.142 | 1.025 (0.776, 1.355) | 0.860 | -0.020 | 0.141 | 0.980 (0.743, 1.292) | 0.886 | -0.003 | 0.095 | 0.997 (0.827, 1.202) | 0.977 |
|  | SVS | 43 | -0.136 | 0.107 | 0.873 (0.741, 1.027) | 0.204 | -0.213 | 0.139 | 0.809 (0.616, 1.062) | 0.126 | -0.205 | 0.133 | 0.815 (0.628, 1.057) | 0.123 | -0.136 | 0.107 | 0.873 (0.707, 1.077) | 0.211 |
|  | Longevity90 | 42 | -0.253 | 0.072 | 0.777 (0.674, 0.895) | **4.620E-04** | -0.180 | 0.115 | 0.835 (0.666, 1.047) | 0.119 | -0.194 | 0.105 | 0.824 (0.670, 1.013) | 0.066 | -0.253 | 0.059 | 0.777 (0.692, 0.871) | 9.669E-05 |
| Vitamin K1 | CAD | 8 | -0.015 | 0.017 | 0.985 (0.953, 1.018) | 0.368 | -0.025 | 0.029 | 0.975 (0.921, 1.032) | 0.386 | -0.021 | 0.021 | 0.979 (0.939, 1.020) | 0.313 | -0.015 | 0.008 | 0.985 (0.969, 1.001) | 0.107 |
|  | MI | 8 | -0.005 | 0.019 | 0.995 (0.958, 1.033) | 0.798 | -0.032 | 0.035 | 0.969 (0.905, 1.037) | 0.360 | -0.023 | 0.026 | 0.977 (0.929, 1.027) | 0.361 | -0.005 | 0.016 | 0.995 (0.964, 1.028) | 0.774 |
|  | AF | 11 | 0.007 | 0.012 | 1.007 (0.984, 1.031) | 0.558 | 0.004 | 0.018 | 1.004 (0.970, 1.039) | 0.813 | 0.004 | 0.015 | 1.004 (0.975, 1.035) | 0.768 | 0.007 | 0.004 | 1.007 (1.000, 1.014) | 0.089 |
|  | HF | 10 | 0.043 | 0.014 | 1.044 (1.015, 1.074) | **0.003** | 0.006 | 0.026 | 1.006 (0.956, 1.059) | 0.820 | 0.014 | 0.021 | 1.014 (0.972, 1.057) | 0.525 | 0.043 | 0.017 | 1.044 (1.009, 1.080) | 0.036 |
|  | AIS | 8 | 0.060 | 0.094 | 1.062 (0.936, 1.205) | 0.522 | 0.179 | 0.145 | 1.196 (0.900, 1.590) | 0.216 | 0.151 | 0.095 | 1.162 (0.964, 1.402) | 0.115 | 0.060 | 0.094 | 1.062 (0.883, 1.277) | 0.543 |
|  | AS | 9 | 0.040 | 0.019 | 1.041 (1.004, 1.080) | **0.031** | 0.024 | 0.037 | 1.024 (0.951, 1.102) | 0.528 | 0.036 | 0.025 | 1.037 (0.988, 1.088) | 0.140 | 0.040 | 0.014 | 1.041 (1.012, 1.070) | 0.022 |
|  | CES | 9 | -0.049 | 0.040 | 0.952 (0.880, 1.029) | 0.217 | -0.080 | 0.067 | 0.923 (0.810, 1.051) | 0.227 | -0.063 | 0.052 | 0.939 (0.848, 1.040) | 0.228 | -0.049 | 0.039 | 0.952 (0.882, 1.028) | 0.242 |
|  | LAS | 9 | 0.158 | 0.054 | 1.172 (1.054, 1.302) | **0.003** | 0.222 | 0.099 | 1.248 (1.027, 1.516) | **0.026** | 0.210 | 0.070 | 1.233 (1.075, 1.416) | **0.003** | 0.158 | 0.036 | 1.172 (1.091, 1.258) | 0.002 |
|  | SVS | 9 | -0.091 | 0.047 | 0.913 (0.833, 1.001) | 0.053 | 0.007 | 0.089 | 1.007 (0.846, 1.200) | 0.935 | -0.091 | 0.062 | 0.913 (0.808, 1.031) | 0.143 | -0.091 | 0.037 | 0.913 (0.849, 0.982) | 0.040 |
|  | Longevity90 | 11 | -0.062 | 0.035 | 0.940 (0.877, 1.008) | 0.082 | -0.082 | 0.057 | 0.922 (0.824, 1.031) | 0.152 | -0.073 | 0.047 | 0.930 (0.847, 1.020) | 0.123 | -0.062 | 0.032 | 0.940 (0.882, 1.002) | 0.086 |

Note: *p*-values below the threshold of 0.05 are displayed in bold.

**Supplementary Table 4. Sensitivity analyses of the Mendelian randomization study.**

|  |  |  |  | **Cochrane’s Q** | | **MR Egger** | | | | **MRPRESSO** | | | | **MRPRESSO_out_adjust** | |
| --- | --- | --- | --- | --- | --- | --- | --- | --- | --- | --- | --- | --- | --- | --- | --- |
| **Exposures** | **Outcomes** | **PVE** | **F_min** | **Heter.Stat** | **Heter.*p*** | **Slope OR(95%CI)** | ***p*-value** | **Intercept (95%CI)** | ***p*-value** | **Heter.est** | **Heter. *p*-value** | **global.rssobs** | **global.*p*** | **OR(95%CI)** | ***p*-value** |
| Calcium | CAD | 0.060 | 19.100 | 665.368 | 1.015E-11 | 0.979 (0.834, 1.148) | 0.789 | 0.002 (-0.001, 0.005) | 0.260 | 665.368 | 0.000 | 667.993 | <1E-04 | 1.067 (0.997, 1.142) | 0.060 |
|  | MI | 0.059 | 19.100 | 606.473 | 6.667E-08 | 1.027 (0.865, 1.220) | 0.758 | 0 (-0.003, 0.004) | 0.853 | 606.473 | 0.000 | 608.861 | <1E-04 |  |  |
|  | AF | 0.059 | 19.100 | 685.478 | 1.311E-12 | 0.992 (0.890, 1.106) | 0.891 | 0 (-0.003, 0.002) | 0.760 | 685.478 | 0.000 | 688.058 | <1E-04 | 0.975 (0.929, 1.022) | 0.294 |
|  | HF | 0.058 | 19.100 | 505.539 | 0.012 | 0.872 (0.777, 0.978) | **0.020** | 0.003 (0, 0.005) | **0.025** | 505.539 | 0.012 | 507.589 | **0.011** |  |  |
|  | AIS | 0.049 | 19.100 | 358.083 | 0.784 | 1.046 (0.624, 1.751) | 0.865 | 0.002 (-0.008, 0.012) | 0.667 | 358.083 | 0.784 | 359.730 | 0.787 |  |  |
|  | AS | 0.060 | 19.100 | 518.219 | 0.004 | 0.985 (0.860, 1.127) | 0.821 | 0.001 (-0.002, 0.004) | 0.565 | 518.219 | 0.004 | 520.204 | **0.004** |  |  |
|  | CES | 0.062 | 19.100 | 461.504 | 0.308 | 1.015 (0.780, 1.321) | 0.912 | -0.001 (-0.007, 0.004) | 0.620 | 461.504 | 0.308 | 463.194 | 0.317 |  |  |
|  | LAS | 0.060 | 19.100 | 433.451 | 0.592 | 1.020 (0.720, 1.446) | 0.910 | -0.002 (-0.009, 0.006) | 0.675 | 433.451 | 0.592 | 435.082 | 0.593 |  |  |
|  | SVS | 0.069 | 19.100 | 486.558 | 0.080 | 0.968 (0.749, 1.253) | 0.806 | 0.001 (-0.005, 0.007) | 0.714 | 486.558 | 0.080 | 488.086 | 0.084 |  |  |
|  | Longevity90 | 0.058 | 19.100 | 372.154 | 0.983 | 0.834 (0.634, 1.099) | 0.197 | 0.001 (-0.005, 0.006) | 0.779 | 372.154 | 0.983 | 373.621 | 0.983 |  |  |
| Vitamin D | CAD | 0.050 | 20.592 | 31.876 | 0.913 | 0.982 (0.894, 1.079) | 0.709 | 0.001 (-0.007, 0.009) | 0.820 | 31.876 | 0.913 | 32.829 | 0.919 |  |  |
|  | MI | 0.048 | 20.592 | 25.366 | 0.974 | 0.953 (0.853, 1.065) | 0.394 | 0 (-0.01, 0.009) | 0.983 | 25.366 | 0.974 | 26.286 | 0.977 |  |  |
|  | AF | 0.529 | 20.457 | 102.814 | 0.785 | 1.013 (0.989, 1.038) | 0.286 | -0.001 (-0.005, 0.003) | 0.618 | 102.814 | 0.785 | 104.270 | 0.790 |  |  |
|  | HF | 0.075 | 20.592 | 42.513 | 0.363 | 1.020 (0.945, 1.102) | 0.600 | 0.002 (-0.006, 0.009) | 0.614 | 42.513 | 0.363 | 43.433 | 0.419 |  |  |
|  | AIS | 0.021 | 20.592 | 13.268 | 0.973 | 0.932 (0.360, 2.415) | 0.885 | -0.006 (-0.058, 0.045) | 0.816 | 13.268 | 0.973 | 14.445 | 0.973 |  |  |
|  | AS | 0.039 | 20.592 | 22.313 | 0.995 | 1.193 (1.017, 1.399) | **0.030** | -0.009 (-0.021, 0.002) | 0.117 | 22.313 | 0.995 | 23.459 | 0.995 |  |  |
|  | CES | 0.048 | 20.592 | 40.566 | 0.577 | 1.080 (0.818, 1.425) | 0.582 | 0 (-0.021, 0.021) | 0.974 | 40.566 | 0.577 | 42.089 | 0.599 |  |  |
|  | LAS | 0.051 | 20.592 | 48.618 | 0.257 | 1.029 (0.709, 1.493) | 0.879 | -0.003 (-0.033, 0.027) | 0.845 | 48.618 | 0.257 | 50.482 | 0.274 |  |  |
|  | SVS | 0.048 | 20.592 | 69.788 | 0.005 | 0.772 (0.525, 1.135) | 0.183 | 0.012 (-0.019, 0.043) | 0.441 | 69.788 | 0.005 | 71.969 | **0.004** |  |  |
|  | Longevity90 | 0.050 | 20.592 | 26.940 | 0.956 | 0.877 (0.670, 1.147) | 0.339 | -0.011 (-0.032, 0.01) | 0.297 | 26.940 | 0.956 | 28.033 | 0.961 |  |  |
| Vitamin K1 | CAD | 0.080 | 15.985 | 1.659 | 0.976 | 0.967 (0.907, 1.031) | 0.309 | 0.009 (-0.017, 0.035) | 0.518 | 1.659 | 0.976 | 1.991 | 0.979 |  |  |
|  | MI | 0.080 | 15.985 | 5.118 | 0.646 | 0.992 (0.911, 1.081) | 0.838 | 0.001 (-0.032, 0.035) | 0.931 | 5.118 | 0.646 | 6.521 | 0.662 |  |  |
|  | AF | 0.120 | 15.985 | 0.969 | 1.000 | 1.014 (0.970, 1.060) | 0.532 | -0.002 (-0.015, 0.01) | 0.712 | 0.969 | 1.000 | 1.171 | 1.000 |  |  |
|  | HF | 0.106 | 15.985 | 13.088 | 0.159 | 0.963 (0.910, 1.020) | 0.197 | 0.026 (0.01, 0.042) | **0.001** | 13.088 | 0.159 | 16.040 | 0.181 |  |  |
|  | AIS | 0.097 | 20.231 | 14.952 | 0.037 | 0.581 (0.373, 0.905) | **0.024** | 0.137 (0.042, 0.233) | **0.013** | 14.952 | 0.037 | 18.793 | **0.046** |  |  |
|  | AS | 0.104 | 15.985 | 4.678 | 0.791 | 1.002 (0.925, 1.086) | 0.953 | 0.012 (-0.01, 0.034) | 0.302 | 4.678 | 0.791 | 5.868 | 0.804 |  |  |
|  | CES | 0.100 | 15.985 | 7.650 | 0.468 | 0.859 (0.699, 1.056) | 0.125 | 0.031 (-0.025, 0.088) | 0.233 | 7.650 | 0.468 | 9.465 | 0.489 |  |  |
|  | LAS | 0.103 | 15.985 | 3.631 | 0.889 | 1.355 (1.076, 1.706) | **0.010** | -0.04 (-0.097, 0.017) | 0.164 | 3.631 | 0.889 | 4.589 | 0.900 |  |  |
|  | SVS | 0.103 | 15.985 | 4.978 | 0.760 | 0.856 (0.707, 1.037) | 0.112 | 0.019 (-0.031, 0.069) | 0.453 | 4.978 | 0.760 | 6.372 | 0.756 |  |  |
|  | Longevity90 | 0.122 | 15.985 | 8.363 | 0.593 | 1.062 (0.918, 1.228) | 0.421 | -0.037 (-0.077, 0.002) | 0.063 | 8.363 | 0.593 | 9.986 | 0.617 |  |  |

Note: *p*-values below the threshold of 0.05 are displayed in bold.

**Supplementary Table 5. Basic characteristics of different CVDs with serum calcium and vitamin D levels**

| **CVDs** | **Features** | **Total** | **Control** | **Case** |
| --- | --- | --- | --- | --- |
| MI | N | 429863 | 416767 | 13096 |
|  | Age M ± SD | 56.42 ± 8.48 | 56.42 ± 8.10 | 56.40 ± 16.38 |
|  | Male (%) | 198737 (46.2) | 188630 (45.3) | 10107 (75.1) |
|  | Calcium levels M ± SD | 2.38 ± 0.11 | 2.38 ± 0.09 | 2.28 ± 0.36 |
| Stroke | N | 429863 | 421705 | 8158 |
|  | Age M ± SD | 56.42 ± 8.48 | 56.45 ± 8.09 | 55.05 ± 19.67 |
|  | Male (%) | 198737 (46.2) | 193061 (45.8) | 5676 (65.8) |
|  | Calcium levels M ± SD | 2.38 ± 0.11 | 2.38 ± 0.09 | 2.23 ± 0.43 |
| CAD | N | 429863 | 389400 | 40463 |
|  | Age M ± SD | 56.55 ± 8.09 | 56.07 ± 8.09 | 61.15 ± 6.45 |
|  | Male (%) | 198513 (46.2) | 170617 (43.8) | 27896 (68.9) |
|  | Calcium levels M ± SD | 2.38 ± 0.09 | 2.38 ± 0.09 | 2.38 ± 0.10 |
| AF | N | 429863 | 401215 | 28648 |
|  | Age M ± SD | 56.55 ± 8.09 | 56.15 ± 8.08 | 62.18 ± 5.93 |
|  | Male (%) | 198513 (46.2) | 180107 (44.9) | 18406 (64.2) |
|  | Calcium levels M ± SD | 2.38 ± 0.09 | 2.38 ± 0.09 | 2.37 ± 0.10 |
| HF | N | 429863 | 416470 | 13393 |
|  | Age M ± SD | 56.55 ± 8.09 | 56.37 ± 8.08 | 62.15 ± 6.14 |
|  | Male (%) | 198513 (46.2) | 189789 (45.6) | 8724 (65.1) |
|  | Calcium levels M ± SD | 2.38 ± 0.09 | 2.38 ± 0.09 | 2.37 ± 0.10 |
| MI | N | 448277 | 434642 | 13635 |
|  | Age M ± SD | 56.34 ± 8.51 | 56.34 ± 8.13 | 56.48 ± 16.38 |
|  | Male (%) | 208424 (46.5) | 197788 (45.5) | 10636 (75.9) |
|  | Vitamin D levels M± SD | 48.52 ± 21.20 | 48.66 ± 21.10 | 44.19 ± 23.75 |
| Stroke | N | 448277 | 439755 | 8522 |
|  | Age M ± SD | 56.34 ± 8.51 | 56.37 ± 8.12 | 55.14 ± 19.65 |
|  | Male (%) | 208424 (46.5) | 202453 (46.1) | 5971 (66.2) |
|  | Vitamin D levels M± SD | 48.52 ± 21.20 | 48.65 ± 21.10 | 42.34 ± 24.79 |
| CAD | N | 448277 | 405972 | 42305 |
|  | Age M ± SD | 56.47 ± 8.12 | 55.98 ± 8.12 | 61.23 ± 6.42 |
|  | Male (%) | 208194 (46.4) | 178802 (44.0) | 29392 (69.5) |
|  | Vitamin D levels M± SD | 48.61 ± 21.11 | 48.72 ± 21.08 | 47.62 ± 21.40 |
| AF | N | 448277 | 418009 | 30268 |
|  | Age M ± SD | 56.47 ± 8.12 | 56.06 ± 8.10 | 62.21 ± 5.93 |
|  | Male (%) | 208194 (46.4) | 188607 (45.1) | 19587 (64.7) |
|  | Vitamin D levels M± SD | 48.61 ± 21.11 | 48.58 ± 21.10 | 49.11 ± 21.25 |
| HF | N | 448277 | 434204 | 14073 |
|  | Age M ± SD | 56.47 ± 8.12 | 56.29 ± 8.11 | 62.23 ± 6.10 |
|  | Male (%) | 208194 (46.4) | 198950 (45.8) | 9244 (65.7) |
|  | Vitamin D levels M± SD | 48.61 ± 21.11 | 48.69 ± 21.09 | 46.29 ± 21.71 |

N, number; M ± SD, mean ± standard deviation
