# Supplementary figures and images for: The genetic correlation and causal association between key factors that influence vascular calcification and cardiovascular disease incidence
Source: Front Cardiovasc Med. 2023 Jan 26;10:1096662. doi: 10.3389/fcvm.2023.1096662 (PMC9908996; doi:10.3389/fcvm.2023.1096662)

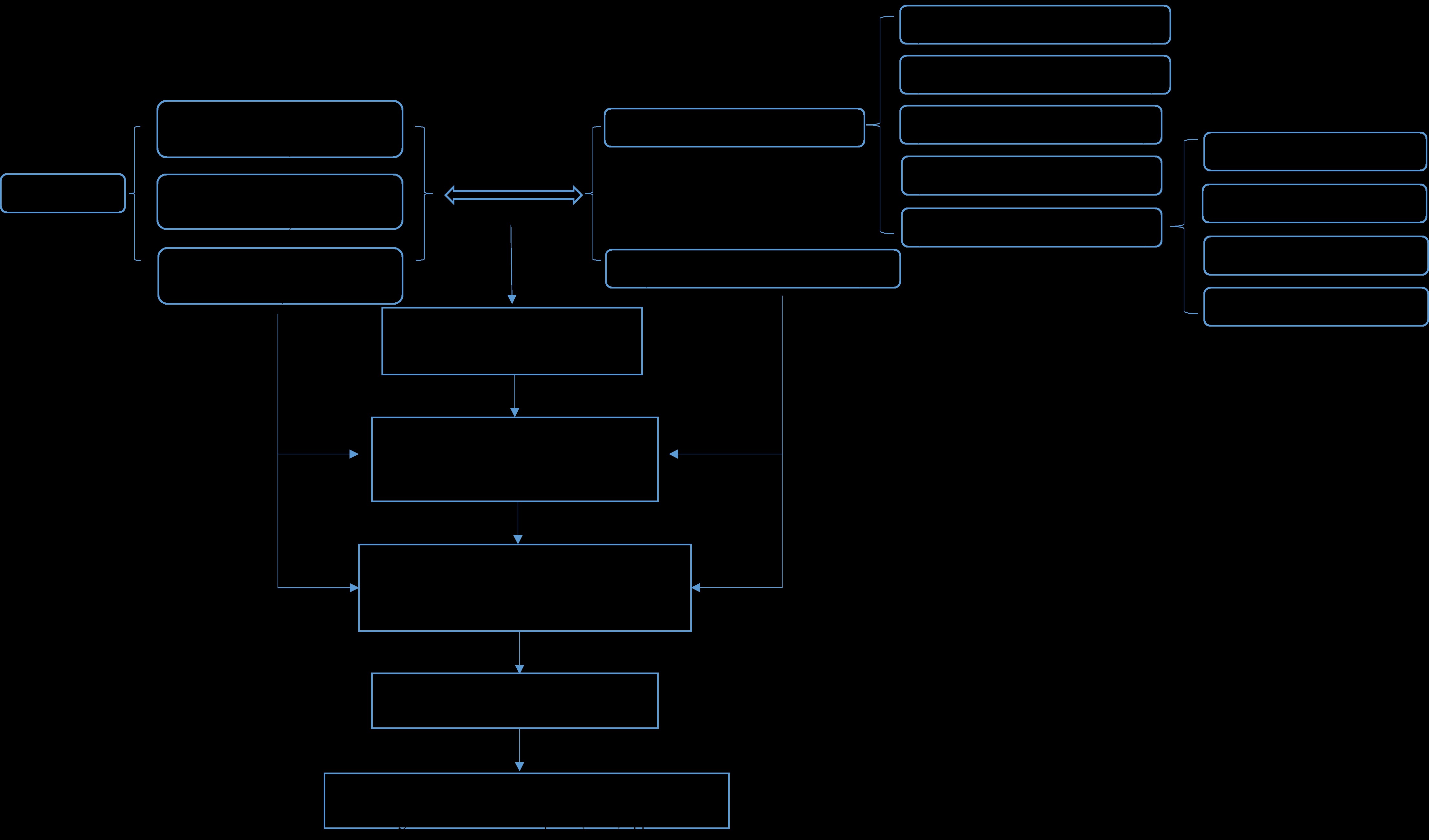

Supplement: Supplementary file 2 [file Image_1.JPEG]

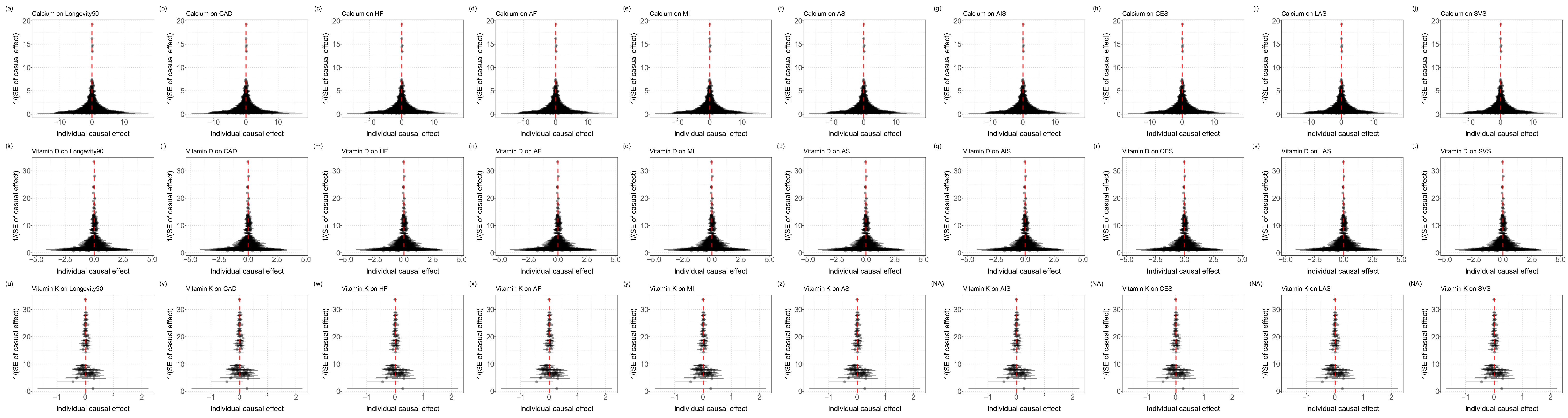

Supplement: Supplementary file 3 [file Image_2.JPEG]

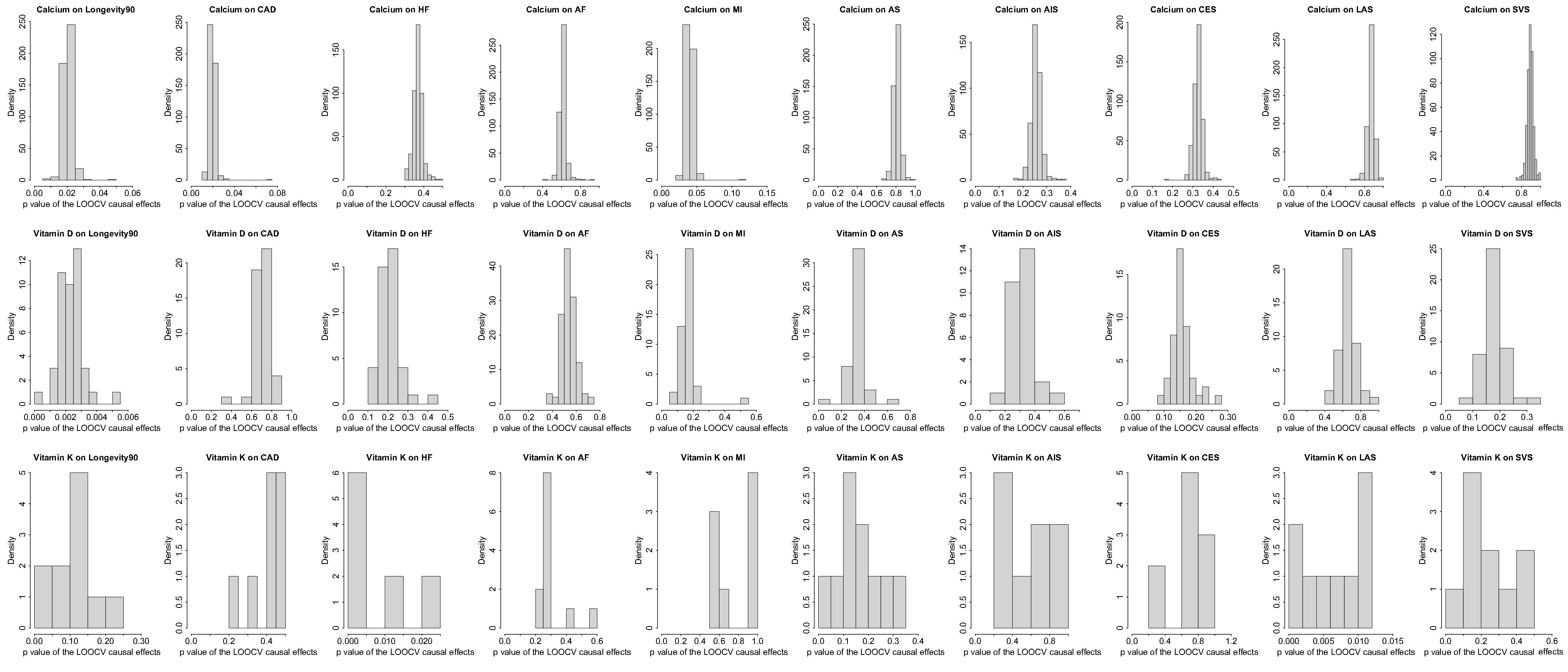

Supplement: Supplementary file 4 [file Image_3.JPEG]
